# Expression of the cellular prion protein by mast cells in the human carotid body

**DOI:** 10.1080/19336896.2023.2193128

**Published:** 2023-03-21

**Authors:** Gregory D. Sweetland, Connor Eggleston, Jason C. Bartz, Candace K. Mathiason, Anthony E. Kincaid

**Affiliations:** a Department of Pharmacy Sciences, School of Pharmacy and Health Professions, Creighton University, Omaha, NE, USA; b Department of Medical Microbiology and Immunology, School of Medicine, Creighton University, Omaha, NE, USA; c Department of Microbiology, Immunology and Pathology, College of Veterinary Medicine and Biomedical Sciences, Colorado State University, Fort Collins, CO, USA

**Keywords:** Carotid body, dorsal motor nucleus of the vagus, glossopharyngeal nerve, intermediolateral cell column, mast cell, neurofilament, neuroinvasion, nucleus of the solitary tract, prion protein, synaptophysin

## Abstract

Prion diseases are fatal neurologic disorders that can be transmitted by blood transfusion. The route for neuroinvasion following exposure to infected blood is not known. Carotid bodies (CBs) are specialized chemosensitive structures that detect the concentration of blood gasses and provide feedback for the neural control of respiration. Sensory cells of the CB are highly perfused and densely innervated by nerves that are synaptically connected to the brainstem and thoracic spinal cord, known to be areas of early prion deposition following oral infection. Given their direct exposure to blood and neural connections to central nervous system (CNS) areas involved in prion neuroinvasion, we sought to determine if there were cells in the human CB that express the cellular prion protein (PrP^C^), a characteristic that would support CBs serving as a route for prion neuroinvasion. We collected CBs from cadaver donor bodies and determined that mast cells located in the carotid bodies express PrP^C^ and that these cells are in close proximity to blood vessels, nerves, and nerve terminals that are synaptically connected to the brainstem and spinal cord.

## Introduction

Prion diseases are a group of fatal neurologic disorders that affect animals including humans. The causative agent is a misfolded version of the endogenous prion protein (PrP^C^) that causes PrP^C^ to change its conformation to a relatively insoluble, protease-resistant version of the prion protein (PrP^Sc^) that tends to form aggregates, that in turn are infectious [1]. The accumulation of PrP^Sc^ in the central nervous system (CNS) results in spongiform degeneration and neuronal death; to date there are no effective treatments [2]. The transmission and progression of prion diseases requires the presence of PrP^C^, a cell surface glycoprotein normally expressed in the CNS; the absence of PrP^C^ prevents prion formation and subsequent disease [3–5]. Following an extraneural route of infection, PrP^Sc^ must enter the CNS for the disease to progress. Transfusion of prion-infected blood is known to transmit disease in sheep [6–9] deer [10–12] and humans [13–15], but the route of entry of the infectious prions into the CNS is not known.

Human carotid bodies (CBs) are small sensory structures (roughly 2-7 mm length by 1.5-4 mm width by 1-2 mm thick) located bilaterally near the bifurcation of the common carotid artery where it terminates by dividing into the internal and external carotid arteries [16–18]. There is some variability in the morphology of the CBs but most commonly they are oval clusters of chemosensitive cells (known as type I cells) surrounded by elongated support cells (known as type II cells). Functionally, type I chemosensitive cells in the CBs are responsive to decreased levels of O_2_ in the blood, but also to increased levels of CO_2_ and low pH. They play an integral role in the adjustment of respiratory rate to meet the changing functional demands of the host [for review see [19]. The clusters of cells are organized into lobules separated by connective tissue septa containing a rich supply of small blood vessels and nerves [20–22]. A characteristic feature of mammalian CBs is their profuse blood supply; they are considered one of the most densely perfused structures in the body based on concentration of blood vessels, a preponderance of fenestrated capillaries and high blood flow rate [23–25]. The robust innervation of the CBs is supplied predominantly by the carotid sinus nerve, an afferent branch of the glossopharyngeal nerve, and to a lesser extent by efferent fibres from sympathetic postganglionic neurons [26–29], for review see [30,31]. It is worth noting that the central processes of the sensory nerves terminate primarily in the nucleus of the solitary tract [NTS [32], and the sympathetic postganglionic neurons located in the superior cervical ganglion that innervate the carotid bodies are known to be synaptically linked to the sympathetic preganglionic neurons located in the intermediolateral cell column [IML] of the thoracic spinal cord., Both of these areas are known to be early sites of PrP^Sc^ accumulation following oral inoculation [33]. Moreover, the dorsal motor nucleus of the vagus [DMNV], which lies adjacent to the NTS in the medulla and is synaptically connected to it [34], is another brainstem area known to be an early site of PrP^Sc^ accumulation following oral inoculation [33].

The goals of this study were to determine if human CBs contain cells that express PrP^C^ and if so, determine if the PrP^C^-expressing cells are located in proximity to neural elements that could provide a route for neuroinvasion. CBs were collected from human donor bodies used in dissection-based anatomy courses at Creighton University. The tissue blocks were embedded in paraffin, sectioned with a microtome and processed for the presence of PrP^C^ and neural markers that are consistent with known routes of prion neuroinvasion.

## Results

**Figure 1.**
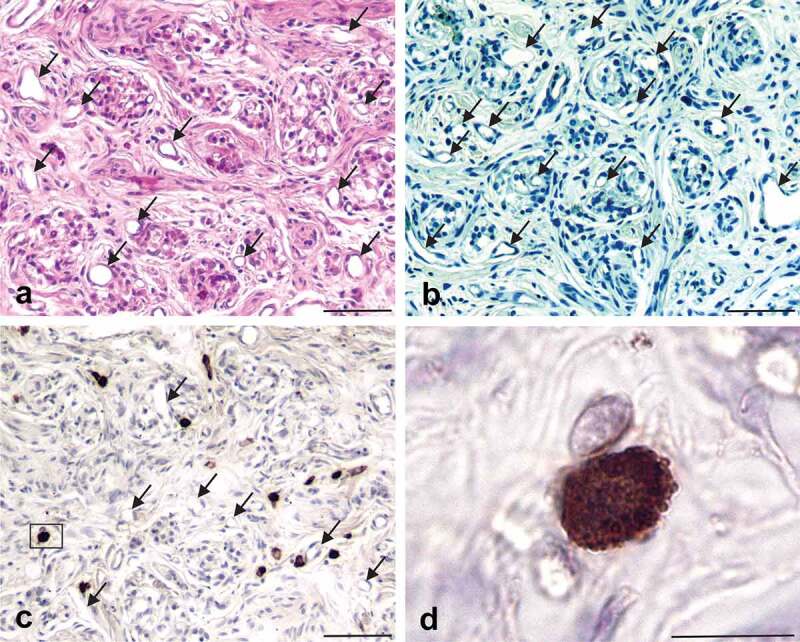
Human CBs contain cells that express PrP^C^. CBs are identified with haematoxylin and eosin (Panel a) and toluidine blue (Panel b) and consist of clusters of cells separated by bands of connective tissue. Note the large number of blood vessels that supply the clusters of cells; lumens of some blood vessels are indicated by arrows. Cells in the CBs that express PrP^C^ are not Type I or Type II cells, but instead are relatively large oval or oblong cells scattered throughout the CB and located primarily in the connective tissue that surrounds the CB lobules (Panel c); lumen of some blood vessels are indicated by arrows. A single PrP^C^-expressing cell (inside the box in Panel c) is enlarged in Panel d and is seen to have the morphology and dense granular appearance that is characteristic of mast cells; lumens of some blood vessels are indicated by arrows. Scale bars: Panel a,b, c = 100 µm; Panel d = 10 µm.

**Figure 2.**
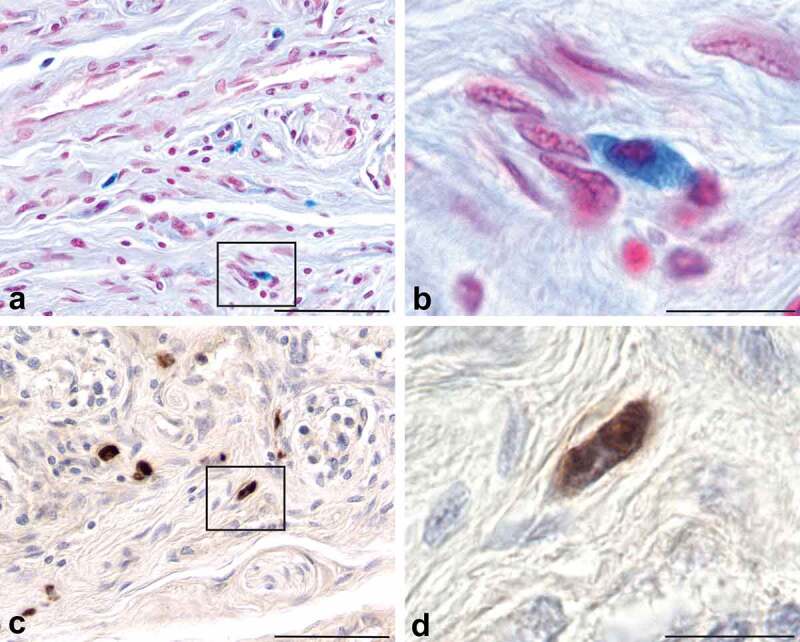
Mast cells in connective tissue septa of CBs can be identified with alcian blue/nuclear fast red stain (Panels a, b) or an antibody against mast cell tryptase (Panels c, d). Note the distribution, morphology, and size of cells stained with alcian blue is similar to the distribution, morphology, and size of the cells stained with the rabbit polyclonal antibody generated against mast cell tryptase. The cell located in the box of panel a and enlarged in panel B is similar to the cell located in the box of panel c that is enlarged in panel d. The mast cells indicated in this figure possess the same distribution, morphology and size as the PrP^C^-expressing cells shown in panels c and d of figure 1. Scale bars: Panels a, c = 50 µm; Panels b, d = 10 µm.

**Figure 3.**
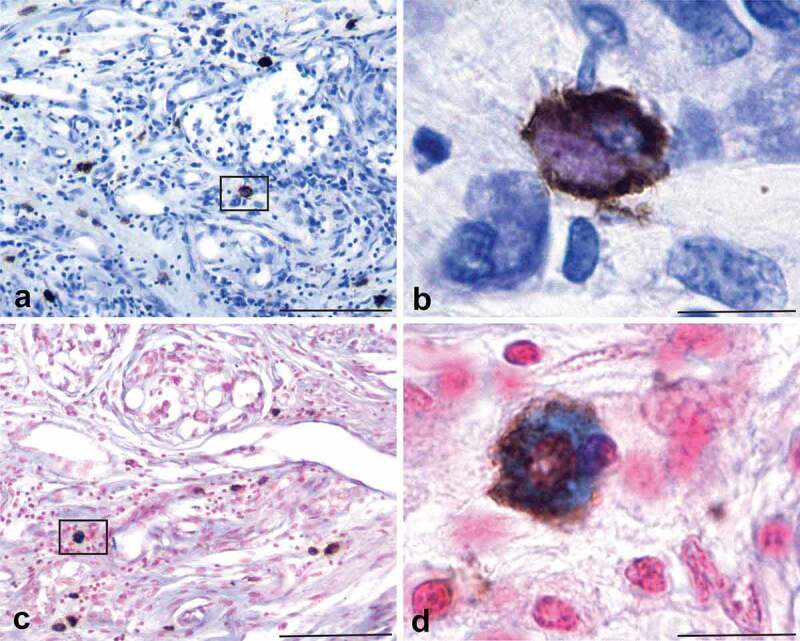
The cells expressing PrP^C^ in the human carotid body are mast cells. Tissue sections of human CB immunohistochemically stained for PrP^C^ expression and counterstained with either TB/ammonium sulphate (Panels a, b) or alcian blue/nuclear fast red (Panels c, d) demonstrate that mast cells express PrP^C^. PrP^C^ expression is indicated by the brown reaction product, while the mast cell phenotype is indicated by purple (panel b) or blue (panel d) staining of the cell cytoplasm. The colocalization of the two markers in the same cell indicate that mast cells express PrP^C^. Scale bars: Panels a, c = 100 µm; Panels b, d = 10 µm.

Carotid bodies (*n* = 29) were collected from 20 donor bodies, sectioned and stained with haematoxylin and eosin (H&E) or toluidine blue (TB). A total of 21 CBs from 14 donor bodies were used in this study. A minimum of 50 tissue sections per CB (Table 1) were processed using a variety of stains and antibodies and examined with a light microscope. Representative photomicrographs were taken of tissue sections containing CBs for each stain or antibody used in each of the CB samples. CBs were easily identified in H&E- or TB-stained tissue sections by their characteristic organization of dense cell clusters, or lobules, supplied by numerous small blood vessels and nerves, and separated by connective tissue septa [Figure 1(a,b)]. Cells in the CB that express PrP^C^ were identified immunohistochemically (IHC) using an antibody (8H4) that recognizes residues 145–180 of the human prion protein [35]. PrP^C^-expressing cells were identified in every CB that was examined; the cells were distributed unevenly throughout the CBs, usually within the connective tissue septa and often near blood vessels (Figure 1c). PrP^C^-expressing cells were granular in appearance, and irregular, fusiform, or oval shaped and varied in diameter from 8–18 µm (Figure 1(c,d)). These cells bore a strong resemblance to mast cells in CBs that were identified using TB/ammonium sulphate, alcian blue/nuclear fast red or an antibody generated against mast cell tryptase (Figures 2a–d) [36]. To determine if PrP^C^-expressing cells associated with CBs were mast cells we combined PrP^C^ IHC with TB or alcian blue counterstaining and empirically adjusted the intensity of chromagen signal and mast cell staining intensity so that both markers could be visualized on the same tissue section (Figure 3). In these tissue sections both the mast cell marker (blue or purple) and the antibody-detected tryptase (brown) can be seen in the same cell (Figure 3(b–d)). It appeared that almost every mast cell located in, or near, the CBs expressed PrP^C^. Thus, we concluded that mast cells express PrP^C^. There was a small number of mast cells (0–2 per tissue section) that were not PrP^C^ immunoreactive in these CBs, but it was not possible to tell if this was due to a technical issue, or if in fact there exists a small population of mast cells in the CB that do not express PrP^C^.

**Table 1.** Donor body information and tissue collection.

Donor	Age	Sex	Carotid bodies analyzed/# of tissue sections examined
C-157	85	Female	left/(66)
C-211	84	Female	right/106 & left/80
C-199	73	Female	right/116 & left/78
A-194	64	Male	right/88 & left/114
A-69	91	Female	right/60 & left/126
C-81	89	Female	right/84 & left/62
C-116	81	Male	right/102 & left/82
C-203	68	Male	right/122 & left/84
A-231	92	Female	left/68
C-222	84	Female	left/50
C-101	80	Female	left/124
C-133	92	Female	right/74
C-118	88	Male	right/52
A-23	86	Male	left/70

**Figure 4.**
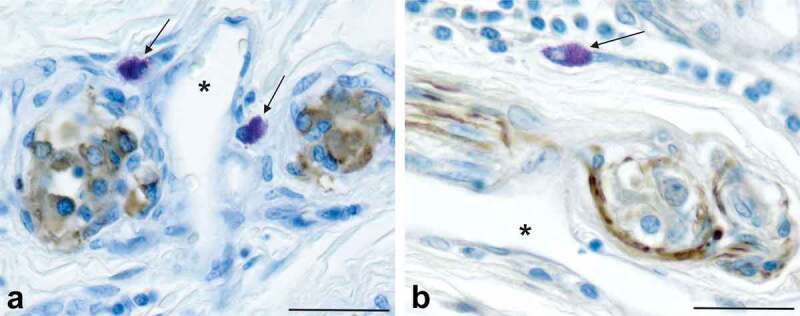
PrP^C^-expressing mast cells in the human carotid body are located within microns of blood vessels (indicated by asterisks) and neural elements (stained brown). Mast cells that express PrP^C^ can be seen near type I CB cells that contain presynaptic vesicles stained with an antibody against synaptophysin (panel a) and axons stained with an antibody against neurofilament-l (panel b). Type I cells in the CB are innervated by peripheral nerve terminals of the carotid sinus nerve; most of the axons located in the CB are components of the carotid sinus nerve. The carotid sinus nerve terminates in the nucleus of the solitary tract in the brainstem, a known clinical target area for prion neuroinvasion. The proximity of the PrP^C^-expressing cells, permeable blood vessels and neural elements is consistent with the potential for prion neuroinvasion when there is a prionemia. Scale bars: Panels a, b = 20 µm.

A set of tissue sections from each donor body was processed to identify neural elements and mast cells in the CB to determine if PrP^C^-expressing cells were in the vicinity of cellular structures synaptically linked to CNS areas known to be sites of prion accumulation following inoculation. This was accomplished using an antibody generated against either synaptophysin, a protein found in presynaptic vesicles [37], or neurofilament-L, an intermediate filament found in axons [38,39], and counterstaining the sections with TB/ammonium sulphate. As shown previously [40] type I CB cells are positive for synaptophysin, which is consistent with their role as chemosensory cells that have vesicles containing neurotransmitter that when released, stimulate sensory terminals [21,41,42]. PrP^C^-expressing mast cells were often located near (within 5–20 µm) synaptic terminals, axons and blood vessels in the CBs (Figure 4(a,b)). Thus, CBs have all the elements required for prion neuroinvasion: exposure to the infectious agent, PrP^C^-expressing cells and proximity to neural elements necessary for transport of prions into the CNS. Although this potential route of neuroinvasion involves the presence of prions in blood it avoids the blood brain barrier and does not utilize brain areas known to have a modified blood brain barrier, such as the area postrema or choroid plexus [33].

Omission of the primary or secondary antibody, or replacement of the primary antibody with an isotype control at the same concentration as the primary antibody resulted in a lack of staining for each of the four antibodies utilized, demonstrating the specificity of the reagents and the antibodies used in this study (Supplementary Figure S1).

## Discussion

**Figure 5.**
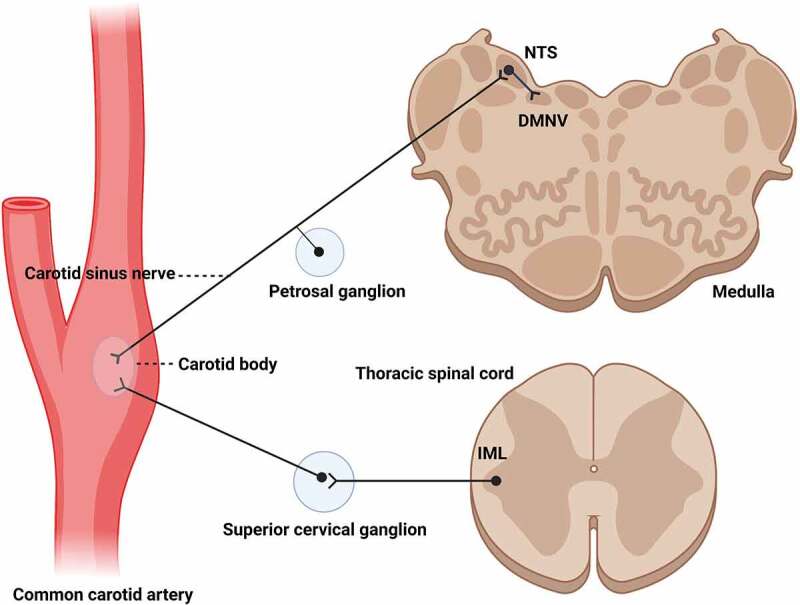
Schematic representation of the potential routes of neuroinvasion following carotid body exposure to prions in blood. The circles represent the location of neuronal cell bodies, the lines represent axons, and the ‘v’ line splits represent axon terminals. The carotid body is located near the bifurcation of the common carotid artery. It is a highly perfused sensory structure that is densely innervated by sensory fibres of the carotid sinus nerve (a branch of the glossopharyngeal nerve) and postganglionic fibres of sympathetic neurons whose cell bodies are located in the superior cervical ganglion. These neural pathways are synaptically linked to areas of the CNS known to be affected early in prion neuroinvasion: the nucleus of the solitary tract (NTS), the dorsal motor nucleus of the vagus (DMNV) and the intermediolateral cell column of the thoracic spinal cord (IML). These structures have been identified as early sites of prion neuroinvasion following oral exposure; note the similarity of this figure to Figure 5 in [33] and Figure 3 in [50]. This figure was created with BioRender.Com.

Mast cells are multifunctional immune cells located in mucosal or connective tissues throughout the body [43,44]. The density and distribution of mast cells reported here is consistent with previous studies reporting mast cells being present in all human CBs, but in varying numbers in different individuals [16,21,45]. The identification of PrP^C^ expression by mast cells in human carotid bodies reported here is consistent with an earlier report of PrP^C^ expression by a human mast cell line [46]. Mast cells tend to be located primarily in the connective tissue bands that separate the clusters of Type I and Type II CB cells. These connective tissue septa contain blood vessels and nerves, and thus mast cells are positioned near blood-borne infectious prions and neural elements that could mediate neuroinvasion of prions into the CNS. It should be noted that there has not been a report of PrP^Sc^ in mast cells of infected animals to date and that mast cell activation is usually the result of an allergic or inflammatory response which are not normally associated with prion infection, but there may be alternative mast cell activation pathways that are activated during prion pathogenesis [47]. In addition to sensory and autonomic fibres from the CBs the NTS the DMNV and the IML of the thoracic spinal cord are also synaptically linked to a variety of other structures, including the tongue, the nasal cavity and the gastrointestinal system, each of which has been identified as a route for prion infection [48–50]. Given that inoculation of prions via the oral, nasal and intravenous routes can result in a prionemia that extends for the duration of the incubation period [51], the innervation of the carotid bodies may be an additional location where infectious prions enter the CNS via routes of neuroinvasion that are very similar to previously reported paths of prion spread [see Figure 5].

## Significance

This is the first report identifying PrP^C^-expressing cells in human CBs. The presence of PrP^C^-expressing cells in CBs is significant in that the CBs are highly perfused by blood and densely innervated by nerve fibres that are directly connected to known targets of prion neuroinvasion. Mast cells expressing PrP^C^ in CBs are likely exposed to infectious prions in those individuals with a prionemia and may be a source of PrP^C^ that is involved in PrP^Sc^ replication which results in prion neuroinvasion via nerves innervating the CBs. Thus, human CBs may be a site of neuroinvasion following transfusion of infected blood and following infection via other routes, including nasal and oral exposure, that result in a prionemia. The identification of PrP^C^-expression by mast cells in human CBs supports a role for mast cells in prion pathogenesis [46,47].

## Materials and methods

Tissue collection: Carotid bodies were collected from embalmed donor bodies used in gross anatomy dissection courses at Creighton University. All aspects of this work were done in accordance with the policies of the Department of Medical Education at Creighton University. The bifurcation of the common carotid artery was located and dissected free of surrounding tissue on both sides of the body. The common carotid artery with the proximal portions of both internal and external carotid arteries attached (about ¼ inch) was dissected and removed. Care was taken to only collect arteries with minimal calcification in the vessel wall (determined by palpation of the vessels), as calcified tissue interferes with cutting tissue sections. When pre-treatment of the tissue blocks using a decalcification solution was used it interfered with the immunohistochemical identification of some proteins in the CB, so this approach was avoided. A total of 21 of CBs from 14 donor bodies were used in this study (Table 1).

Histology and histochemistry: The tissue specimens were embedded in paraffin and sectioned using a rotary microtome (7 µm) and collected on glass slides. Tissue sections containing CBs were identified following standard staining with H&E and TB (Figure 1(a,b)). 2% TB (Fisher; T161–25) in a 5% aluminium sulphate (J.T. Baker; JT4628–01) solution was used to identify mast cells; the metachromatic reaction selectively rendered the mast cell granules a deep shade of purple, which was easily discernible from the blue nuclei of surrounding cells (Figure 4(a,b)). Alcian blue (Sigma; 1.01647.0500) with nuclear fast red (Vector; H-3403) was also used to stain mast cells, resulting in bright blue staining of mast cell granules and red staining of nuclei (Figure 2(a,b)).

Immunohistochemistry: PrP^C^, mast cells, presynaptic vesicles and axons were immunohistochemically identified in human CBs using antibodies generated against PrP^C^, mast cell tryptase, synaptophysin, or neurofilament light chain using standard immunohistochemical methods (see Table 2 for details). Briefly, tissue sections were deparaffinized and treated with 10% formic acid (10 minutes) to facilitate antigen retrieval. Endogenous peroxidase was blocked using 3% hydrogen peroxide in methanol (20 minutes) and non-specific binding was blocked using 10% normal serum in 0.05% Tween in Tris-buffered saline (TTBS; 30 minutes). Following 3 rinses in TTBS tissue sections were incubated in either a prion antibody, a mast cell antibody, a synaptophysin antibody, or a neurofilament antibody in TTBS with 0.3% normal serum at 35° for 24 hours. Following 3 rinses in TTBS the sections were incubated in biotinylated secondary antibody for 1 hour, then placed in avidin-biotin solution (1:200; Vector Laboratories, Burlingame, CA) for 20–30 minutes and then reacted in filtered 0.05% diaminobenzidine tetrachloride (Sigma, St. Louis, MO) with 0.0015% H_2_O_2_ for 10–20 minutes. The sections were rinsed and counterstained with either haematoxylin, TB/ammonium sulphate or alcian blue/nuclear fast red and then dehydrated using alcohols, cleared in xylene and coverslipped with Cytoseal-XYL (Richard Allan Scientific, Kalamazoo, MI).Some tissue sections were processed in an identical manner but with either the primary or secondary antibodies omitted, or with the same concentration of isotype control in place of the primary antibody (Table 2; Supplementary figure S1). The tissue sections were examined using an Olympus B× 40 light microscope and photographs were taken using a Nikon Eclipse 80i light microscope using ImageJ software.

**Table 2.** Antibodies and isotype controls.

Antibody	Source (catalogue number)	Primary Concentration	Secondary Concentration	Normal Serum	Isotype Control
Prion protein (8H4)	Abcam (61409)	1:500	1:500	Horse	Mouse monoclonal IgG2b
Mast cell tryptase	Abcam (196772)	1:500	1:500	Goat	Rabbit polyclonal IgG
Synaptophysin (Clone SY38)	Dako (0776)	1:500	1:500	Horse	Mouse monoclonal IgG1k
Neurofilament (NF421 + NFl/736)	Abcam (215903)	1:500	1:500	Horse	Mouse monoclonal IgG1

## Supplementary Material

Supplemental Material
