# Supplementary material for: Expression of the cellular prion protein by mast cells in the human carotid body
Source: Prion. 2023 Mar 21;17(1):67–74. doi: 10.1080/19336896.2023.2193128 (PMC10038025; doi:10.1080/19336896.2023.2193128)
Supplement: Supplemental Material [file KPRN_A_2193128_SM9520.zip › supplementary figure caption.docx]

Supplemental Figure 1: Omission of primary or secondary antibodies, or replacement of primary antibody with the same concentration of isotype control results in a lack of staining in CB tissue sections taken from the same donor body and processed at the same time using the same reagents. Panels A-D: 8H4 antibody that recognizes PrP^C^ identifies a population of PrP^C^-expressing cells in the human CB (A); staining of the cells is not observed when the primary (B) or secondary antibody (C) is omitted or when the primary antibody is replaced by an isotype control (D). Panels E-H: An antibody that recognizes mast cell tryptase identifies mast cells in the human CB (E); staining of the cells is not observed when the primary (F) or secondary antibody (G) is omitted or when the primary antibody is replaced by an isotype control (H). Panels I-L: An antibody that recognizes synaptophysin identifies synapses in the human CB (I); staining of the synapses is not observed when the primary (J) or secondary antibody (K) is omitted or when the primary antibody is replaced by an isotype control (L). Panels M-P: An antibody that recognizes neurofilaments identifies axons in the human CB (M); staining of axons is not observed when the primary (N) or secondary antibody (O) is omitted or when the primary antibody is replaced by an isotype control (P). All images photographed at the same magnification. Scale bar Panel A = 50µm.
