# Supplementary figures and images for: Expression of the cellular prion protein by mast cells in the human carotid body
Source: Prion. 2023 Mar 21;17(1):67–74. doi: 10.1080/19336896.2023.2193128 (PMC10038025; doi:10.1080/19336896.2023.2193128)

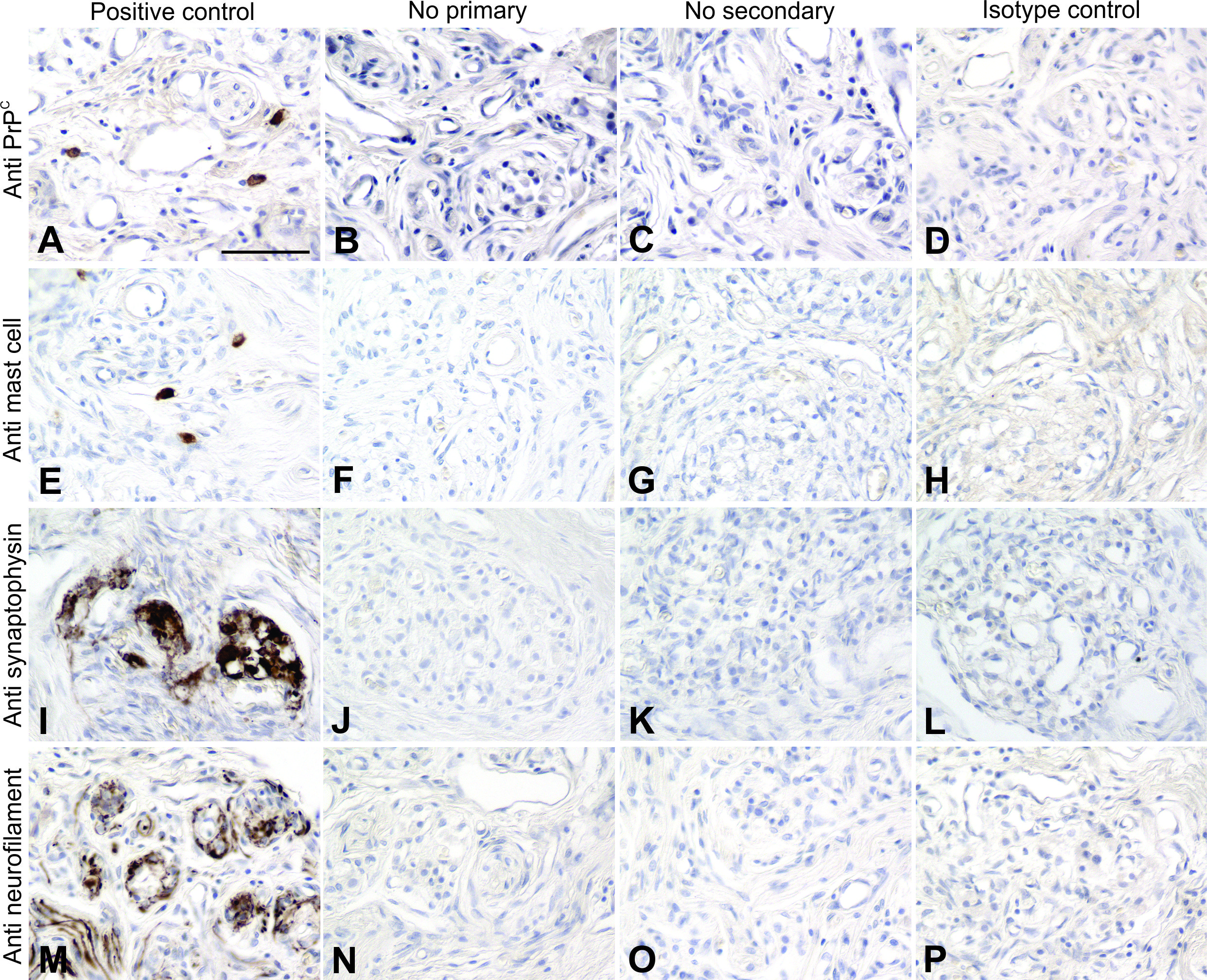

Supplement: Supplemental Material [file KPRN_A_2193128_SM9520.zip › Supplementary Figure 1 (3).jpg]
